# New Roles for Two-Component System Response Regulators of *Salmonella enterica* Serovar Typhi during Host Cell Interactions

**DOI:** 10.3390/microorganisms8050722

**Published:** 2020-05-13

**Authors:** Claudie Murret-Labarthe, Maud Kerhoas, Karine Dufresne, France Daigle

**Affiliations:** Département de microbiologie, infectiologie, immunologie, Université de Montréal, Montréal, QC H3T 1J4, Canada; c.m.labarthe@gmail.com (C.M.-L.); maud1693@hotmail.com (M.K.); karine.dufresne@umontreal.ca (K.D.)

**Keywords:** *Salmonella* Typhi, two-component system, *cpxR*

## Abstract

In order to survive external stresses, bacteria need to adapt quickly to changes in their environment. One adaptive mechanism is to coordinate and alter their gene expression by using two-component systems (TCS). TCS are composed of a sensor kinase that activates a transcriptional response regulator by phosphorylation. TCS are involved in motility, virulence, nutrient acquisition, and envelope stress in many bacteria. The pathogenic bacteria *Salmonella enterica* serovar Typhi (*S.* Typhi) possess 30 TCSs, is specific to humans, and causes typhoid fever. Here, we have individually deleted each of the 30 response regulators. We have determined their role during interaction with host cells (epithelial cells and macrophages). Deletion of most of the systems (24 out of 30) resulted in a significant change during infection. We have identified 32 new phenotypes associated with TCS of *S.* Typhi. Some previously known phenotypes associated with TCSs in *Salmonella* were also confirmed. We have also uncovered phenotypic divergence between *Salmonella* serovars, as distinct phenotypes between *S.* Typhi and *S.* Typhimurium were identified for *cpxR*. This finding highlights the importance of specifically studying *S.* Typhi to understand its pathogenesis mechanisms and to develop strategies to potentially reduce typhoid infections.

## 1. Introduction

Bacteria possess a variety of systems that enable them to respond to diverse signals received from the external environment. These signals are mainly detected by two-component systems (TCS) composed of a histidine sensor kinase (SK) and a response regulator (RR). Physical or chemical signals, such as changes in extracellular ion concentrations, pH, oxygen, osmolarity, quorum sensing, and the presence of antibiotics are some of the signals detected by TCS. TCS are involved in adaptation to several conditions, notably stress conditions, host–pathogen interactions, symbiotic interactions, and intracellular signaling [1,2].

The SK partner of the TCS is located in the inner membrane and generally comprises two domains, a receiver and a transmitter domain that contains a kinase activity with a conserved histidine residue. Typically, the RR proteins are located in the cytoplasm and also comprise two domains, a receiver domain in the N-terminal section of the protein containing a conserved aspartate residue and a response domain in the C-terminal of the protein. When a signal is detected by the SK, this results in autophoshorylation of the conserved histidine residue, an ATP-dependent process. The SK then activates the RR through transfer of its phosphorylated group to the conserved RR aspartate residue. Once activated, the RR initiates the adaptive transcriptional response, through activation or repression of genes that will adjust the bacterial lifestyle to the conditions encountered [3].

*Salmonella enterica* serovar Typhi (*S.* Typhi) is a human-specific bacterial pathogen and the etiologic agent of the typhoid fever. This disease is common in Africa and Southeast Asia and causes between 11.9 and 26.9 million cases and 128,000 to 216,500 deaths per year [4]. Infection with this pathogen occurs through the ingestion of contaminated food or water. Once ingested, *Salmonella* must first resist stomach acidity [5,6], then reach the small intestine, cross the mucosal barrier of the intestine, and gain access to intestinal epithelial cells. Bacteria can then invade epithelial cells using the type-three secretion system (T3SS) located on *Salmonella* pathogenicity island 1 (SPI-1) [7,8]. *S*. Typhi does not elicit a strong intestinal immune response or inflammation, mainly by producing the Vi capsule [9]. It crosses the intestinal barrier, infects macrophages, and survives within vacuoles by using a second T3SS located on SPI-2 [10,11]. *S.* Typhi then causes a systemic infection by disseminating to deeper tissues including spleen, liver, bone marrow, and gallbladder [12].

Currently, most of our knowledge concerning TCS was obtained from studies done in *Escherichia coli* or *Salmonella enterica* serovar Typhimurium. Thus far, only six TCS have been characterized in *S.* Typhi. Both the EnvZ-OmpR system and the Rcs system activate the expression of the Vi capsule [13,14]. The Rcs system also represses invasion proteins and flagellin [14,15,16,17]. The PhoPQ system regulates the *S.* Typhi-specific CdtB, ClyA, and TaiA toxins [18,19,20], is expressed in typhoid patients [21], and a *phoPQ* deletion was used in a live attenuated *S.* Typhi vaccine [22]. The SsrAB system had no role in survival in macrophages in *S.* Typhi [23]. The CpxAR system is involved in adhesion and invasion of human intestinal epithelial cells and is activated by osmolarity [24]. The QseCB system is activated by several signals, including neurotransmitters (epinephrine and norepinephrine) [25,26], and invasion of epithelial cells increased in a *qseB* mutant of *S.* Typhi [27]. UhpBA regulates glucose-6-phosphate transport [28]. A comparative study of the transcriptional profile performed in *S.* Typhi indicates that UhpA was involved in the sulfur assimilation pathway [29]. Other TCS have not been studied in *S.* Typhi and some TCS have not been investigated in *S.* Typhimurium (CitAB, CreCB, DpiBA, TctED, and TorSR).

As some TCS play a role in *S.* Typhi infection, it is likely that other TCS may have a significant role in different stages of disease by this pathogen. To study the TCS of *S.* Typhi, we have deleted each of the genes encoding RR proteins, since it has been shown that some SK can also activate non-specific RR and complement defects of the specific corresponding SK mutant [30]. Non-polar deletions of genes encoding each RR protein were created by allelic exchange and we evaluated the ability of each mutant to adhere, invade, and replicate in human epithelial cells and to be phagocytosed and survive in human macrophages. This study represents a comprehensive characterization of all *S.* Typhi TCS and identifies a potential role for each of these systems in *S.* Typhi pathogenesis.

## 2. Materials and Methods

### 2.1. Bacterial Strains and Growth Conditions

*S.* Typhi strain ISP1820 was used throughout this study as the main wild-type strain [31]. Strains and plasmids used in this study are listed in Supplementary Tables S1 and S2, respectively. Bacteria were routinely grown overnight in Luria-Bertani (LB) broth, with agitation at 37 °C, unless indicated otherwise. Antibiotic or supplements were added at the following concentration: 34 μg/mL chloramphenicol and 50 μg/mL diaminopimelic acid, when required. Bacterial transformation was performed using the calcium/manganese-based method, as previously described [32].

### 2.2. Chromosomal Deletion of TCS Regulatory Genes

Thirty TCS were identified in the sequenced genome of *S.* Typhi strain CT18 [33] by searching for DNA binding protein and regulator. The non-polar deletion of all the response regulator (RR) encoding genes were obtained by allelic exchange, as described previously [34], using the overlap-extension PCR method [35]. Deletions were confirmed by PCR. The primers used for mutagenesis are listed in Supplementary Table S3.

### 2.3. Interaction with Cultured Human Epithelial Intestinal Cells

The INT-407 (ATCC CCL-6) cells were cultivated in Eagle minimal essential medium (EMEM) (Wisent, St-Bruno, QC, Canada) supplemented with 10% heat-inactivated fetal bovine serum (FBS) (Wisent) and 25 mM HEPES (Wisent, St-Bruno, QC, Canada). The gentamicin protection assay described previously was adapted to 96-well plates and performed at a multiplicity of infection (MOI) of 20 [34]. Bacteria were grown overnight in static condition (low aeration) in LB-NaCl (300 mM) to induce SPI-1 and were added in triplicate. After 90 min, infected cells were washed with phosphate-buffer saline (PBS) and fresh medium supplemented with 50 µg/mL gentamicin was added to kill the extracellular bacteria. Cells were lysed with PBS and 0.1% sodium deoxycholate (PBS-DOC) at 90 min (adhesion), 180 min (invasion), and 18 h (survival) post-infection. Serial dilutions were performed for enumeration of viable colony counts by colony-forming units (CFU/mL). The assay was performed at least three times in triplicate.

### 2.4. Infection of Cultured Macrophages

The THP-1 (ATCC TIB-202) cells were cultivated in RPMI 1640 (Wisent, St-Bruno, QC, Canada) supplemented with 10% heat-inactivated FBS (Wisent), 1 mM sodium pyruvate (Wisent), and 1% MEM non-essential amino acids (Wisent, St-Bruno, QC, Canada). The human monocytes cells were differentiated into macrophages by addition of 10^−7^ M phorbol 12-myristate 13 acetate (Sigma) for 48 h before the infection. Similarly, the RAW264.7 (ATCC TIB-71) murine macrophages were cultivated in Dulbecco’s Modified Eagle’s Medium (DMEM; Wisent, St-Bruno, QC, Canada). The method was adapted to 96-well plates and performed at a MOI of 10 [36]. To obtain a similar number of intracellular bacteria, a MOI of 10 was used for macrophages to compensate for the phagocytic activity. Briefly, following an overnight growth in LB broth, the strains were added in triplicate. After 30 min, infected cells were washed with PBS, treated with gentamicin (50 ug/mL), and lysed with PBS-DOC 0.1% at 30 min (phagocytosis), and 18 h (survival) post-infection, then, serial dilutions were performed for enumeration of viable colony counts (CFU/mL). Each deletion was tested at least three times in triplicate.

### 2.5. Motility Assays

Motility assays were performed in a tube, containing the «Motility Test Medium» (BBL, BD, Mississauga, ON, Canada), in which a solution of 1% of triphenyltetrazolium chloride was added. These agar tubes were inoculated by stabbing the agar with an overnight culture of bacteria. The tubes were then incubated at 37 °C for approximately 18 h, to evaluate the motility of the mutants. For each deletion, this assay was performed at least three times. Motility assays on plates were performed as described previously [37].

## 3. Results

### 3.1. Deletion and Characterization of RR Mutants

We have identified 30 RR genes in the genome of *S.* Typhi and an overview of their putative functions is summarized in Table 1. These TCS were all detected in the genome of the closely related serovar Typhimurium. However, these two serovars have a different host range, and cause distinct disease, suggesting that potential differences between these serovars may involve differences in gene regulation. All RR were deleted individually. Deletion of an internal fragment of each RR was achieved by allelic exchange in *S.* Typhi strain ISP1820. Each marker-less deletion was in frame, to avoid any polar effect. Mutants were characterized for their growth, susceptibility to aminoglycoside, and motility. All mutants had a similar growth curve in LB compared to the wild-type parent strain (data not shown). The *arcA* mutant produced smaller colonies on LB agar. The mutants were all sensitive to gentamicin and most mutants were motile as the wild-type (except for *cheY*, as expected, and *ompR*, which demonstrated a reduced swimming area, 85% of the wild-type, in motility medium).

### 3.2. Adhesion, Invasion, and Replication in Epithelial Cells

Passage through the intestinal epithelial cell barrier is a key step in the pathogenesis of *S.* Typhi. We used infection of epithelial cells to evaluate adhesion, invasion, and replication effects of the TCS mutant of *S.* Typhi in these cell type. The wild-type *S.* Typhi ISP1820 strain was used as the reference control and its isogenic *invA* (SPI-1)/*ssrB* (SPI-2) mutant (here referred as ΔSPIs) were used as a low virulence control, as this strain exhibits impaired host cell entry.

**Figure 1:**
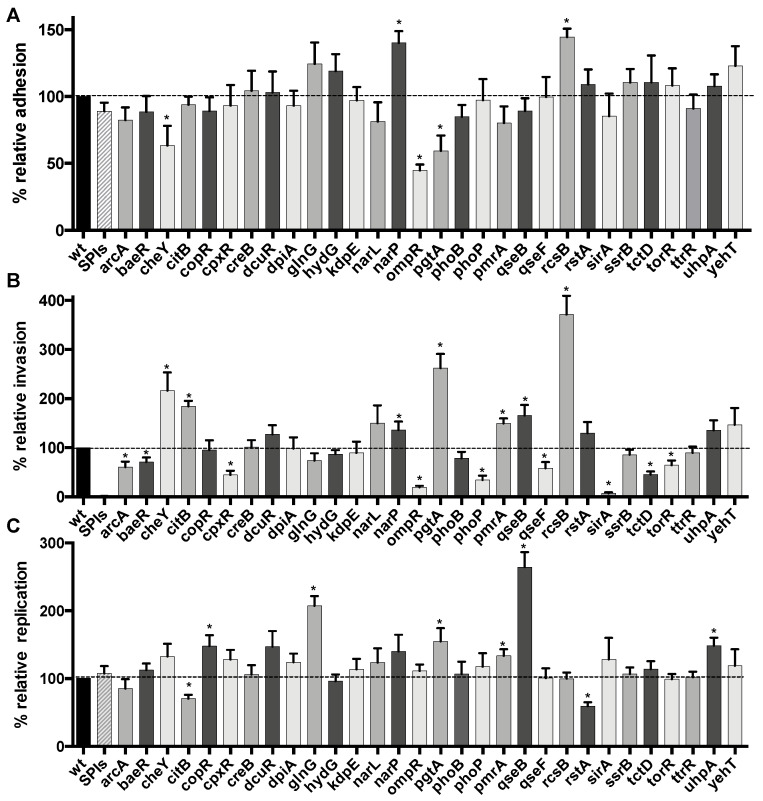
Effect of loss of TCS response regulators on interaction with human epithelial cells. INT-407 epithelial cells were infected with *S*. Typhi wild-type strain and the isogenic RR mutants, and the level of bacteria associated with cells was determined upon adherence (90 min) (**A**), invasion (180 min) (**B**), or after 18 h (**C**). All assays were conducted in triplicate and repeated independently at least three times. The results are expressed as the mean ± SEM of the replicate experiments. Significant differences (* *p* < 0.0001) in the levels recovered as compared to the wild-type were determined by the Student’s unpaired *t*-test. The dashed line corresponds to the wild-type level.

The adhesion level for the different TCS mutants ranged from 45 to 144% of the wild-type (Figure 1A). There were 5 mutants that showed a significant change in adherence compared to the wild-type strain. Three mutants (*cheY*, *ompR* and *pgtA*) were less adherent and 2 mutants (*narP* and *rcsB*) were more adherent. The *ompR* was the least adherent, whereas the *rcsB* mutant had the highest level of cell adherence.

For the cell invasion phenotype, differences in invasion varied from 7 to 370% of the wild-type, and several mutants (16/30) showed a significant difference in cell invasion compared to the wild-type strain. Seven mutants showed increased invasion (*cheY*, *citB*, *narP*, *pgtA*, *pmrA*, *qseB* and *rcsB*) and 9 showed decreased invasion (*arcA*, *baeR*, *cpxR*, *ompR*, *phoP*, *qseF*, *sirA*, *tctD* and *torR*) (Figure 1B). The negative control (ΔSPIs) showed only 1.3% invasion compared to the wild-type, as expected. The TCS mutant demonstrating the most decreased invasion was *sirA* and the mutant with the highest increased invasion was *rcsB*.

For intracellular replication, the range was from 70 to 264% of the wild-type. There were 8 mutants demonstrating significantly different levels of replication, 6 that were higher (*copR*, *glnG*, *pgtA*, *pmrA*, *qseB*, and *uhpA*) and 2 that were lower (*citB* and *rstA*) than the wild-type control (Figure 1C). The *rstA* mutant had the greatest decrease, whereas the *qseB* mutant had the highest level of replication in epithelial cells. Interestingly, several mutants that were defective in invasion were able to replicate similarly to the wild-type.

### 3.3. Uptake and Survival in Macrophages

**Figure 2:**
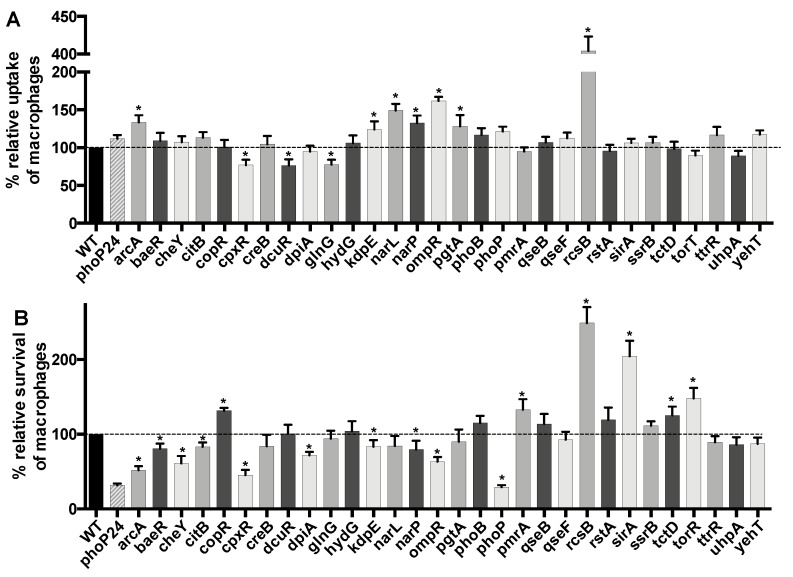
Effect of loss of TCS response regulators during interaction with human macrophages. THP-1 cells were differentiated into macrophages and infected with *S*. Typhi wild-type strain and the isogenic RR mutants. The level of bacterial uptake (phagocytosis) (**A**) and the level of survival after 18 h infection (**B**) were determined. All assays were conducted in duplicate and repeated independently at least three times. The results are expressed as the mean ± SEM of replicate experiments. Significant differences (* *p* < 0.0001) as compared to wild-type were determined by the Student’s unpaired *t*-test. The dashed line corresponds to the wild-type level.

Some TCSs are important for survival of *Salmonella* inside macrophages, and survival within these cells represents a crucial step in the pathogenesis and virulence of *S.* Typhi to disseminate systemically. Thus, we investigated the role of each TCS in uptake and survival in macrophages. The wild-type *S.* Typhi ISP1820 strain was used as the reference control and the *phoP24* isogenic mutant (PhoP constitutive) [108], known to be defective in virulence and macrophages survival [109], was used as a low virulence control. This control was chosen as the isogenic *invA* (SPI-1)/*ssrB* (SPI-2) mutant (ΔSPIs) to survive as the wild-type strain in macrophage [23]. The level of internalization by macrophage varied from between 76 to 404% of the wild-type (Figure 2A). There were 10 mutants with a significant difference in uptake by macrophage compared to the wild-type strain. Seven of the mutants showed increased uptake (*arcA*, *kdpE*, *narL*, *narP*, *ompR*, *pgtA*, and *rcsB*) and three mutants (*cpxR*, *dcuR*, and *glnG*) showed decreased macrophage uptake. The *glnG* mutant demonstrated the lowest level of uptake and the *rcsB* mutant showed the highest level of uptake by macrophage.

The level of survival in macrophages ranged from 29 to 249% of the wild-type (Figure 2B). There were 16 mutants with a significant difference in survival compared to the wild-type strain, 6 showed an increased survival (*copR*, *pmrA*, *rcsB*, *sirA*, *tctD*, and *torR*) and 10 demonstrated a decreased survival (*arcA*, *baeR*, *cheY*, *citB*, *cpxR*, *dpiA*, *kdpE*, *narP*, *ompR*, and *phoP*). The *phoP* mutant demonstrated the lowest survival and the *rcsB* mutant had the highest level of survival in macrophage.

### 3.4. Complementation

**Figure 3:**
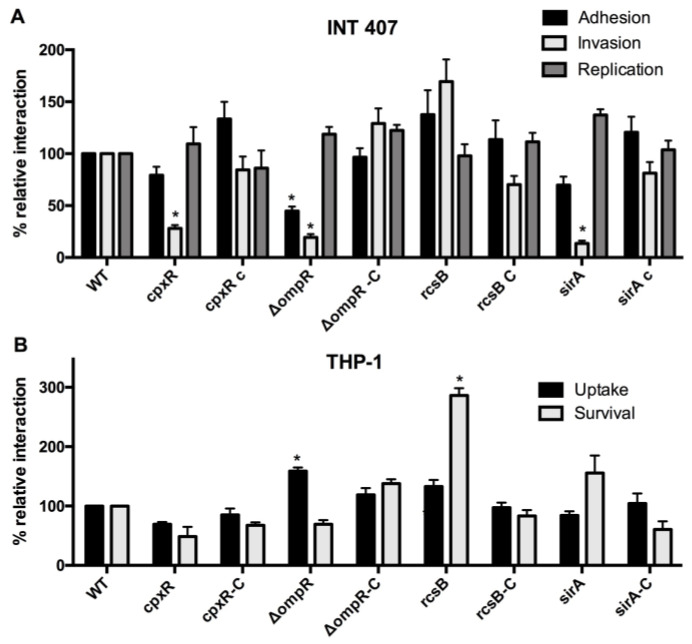
Complementation. Epithelial INT-407 cells (**A**) and THP-1 macrophages (**B**) were infected with *S*. Typhi wild-type strain, the *cpxR, ompR, rcsB*, and *sirA* mutants and complemented mutants with a wild-type copy on a low-copy vector. All assays were conducted in triplicate and repeated independently at least three times. The results are expressed as the mean ± SEM of the replicate experiments. Significant differences (* *p* < 0.0001) in the level between the wild-type and the mutant were determined by the Student’s unpaired *t*-test. The dashed line corresponds to the wild-type level.

In order to confirm that the phenotypic difference was associated with the RR mutation, we selected 4 mutants that were strongly under- or over-represented compared to the wild-type strain in invasion or survival level in macrophages. The *cpxR*, *ompR*, *rcsB*, and *sirA* mutants were complemented with a wild-type copy of the gene on a low-copy vector. Interactions with epithelial cells and macrophages were evaluated. The wild-type levels association with cells were restored in the complemented strains (Figure 3).

### 3.5. Impact of the Vi Antigen

**Figure 4:**
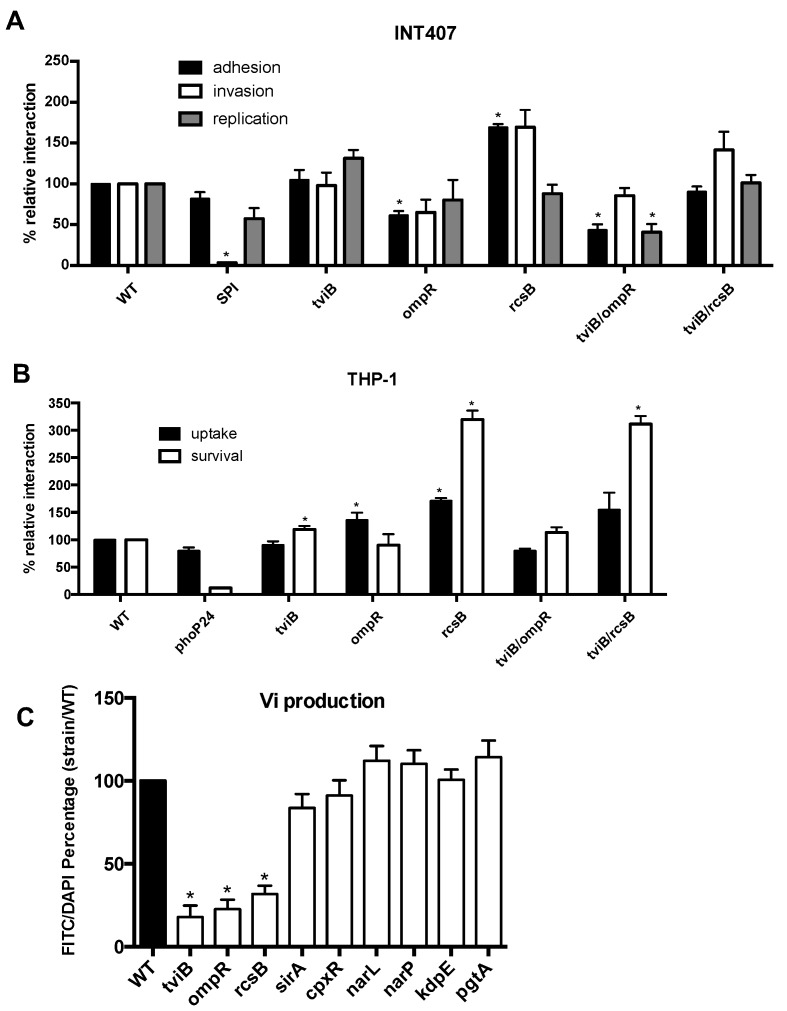
Role of Vi capsule. Epithelial INT-407 cells (**A**) and THP-1 macrophages (**B**) were infected with *S*. Typhi wild-type strain, the *tviB*, *ompR, rcsB* and *the double mutant tviB-ompR* and *tviB-rcsB* mutants. (**C**) Production of the Vi antigen by immuno-staining. All assays were conducted in triplicate and repeated independently at least three times. The results are expressed as the mean ± SEM of the replicate experiments. Significant differences (* *p* < 0.0001) in the level between the wild-type and the mutant were determined by the Student’s unpaired *t*-test.

It was previously demonstrated that RscB and OmpR regulate the Vi capsule [14,15,16,17,19,20]. As these TCS showed strong phenotypes, often opposite, except for phagocytosis, we investigated the role of the Vi antigen during host cell interaction. We have constructed a *tviB* mutant as well as a double *tviB-ompR* and a double *tviB-rcsB* mutant and evaluated these strains with epithelial cells and macrophages (Figure 4). The lower level of adhesion to epithelial cells observed for the *ompR* mutant was specific to *ompR* as the *tviB* mutant was not significantly different than the wild-type, whereas the double *tviB-ompR* was similar to the *ompR* mutant. Similarly, the high level of invasion of epithelial cells observed for the *rcsB* mutant was specific to the *rcsB* mutation as the mutant and the double mutant *tviB-rcsB* were both significantly different than the wild-type but not the *tviB* mutant. The loss of the Vi antigen did not increase the phagocytosis and survival level in macrophages, suggesting that the phenotypes observed were specific to the *ompR* and the *rcsB* mutation. We have confirmed by immuno-staining that the *ompR*, *rcsB*, and *tviB* mutants did not express the Vi antigen compared to the wild-type strain and other mutants (Figure 4C).

### 3.6. Strain Specificity

**Figure 5:**
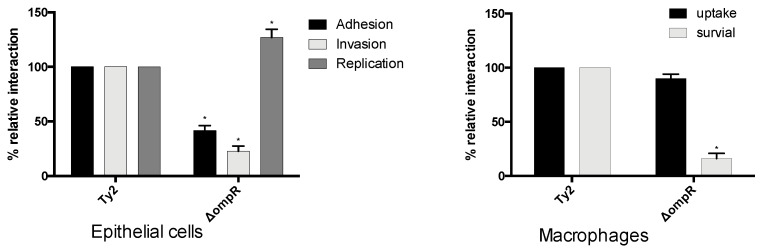
Role of *ompR* mutant in *S.* Typhi strain Ty2. Epithelial INT-407 cells and THP-1 macrophages were infected with *S*. Typhi Ty2 strain and its isogenic *ompR* mutant. All assays were conducted in triplicate and repeated independently at least three times. The results are expressed as the mean ± SEM of the replicate experiments. Significant differences (* *p* < 0.05) compared to the wild-type were determined by the Student’s unpaired *t*-test.

As all mutants were tested in *S.* Typhi strain ISP1820, we also investigated if the *ompR* phenotype was conserved in another *S.* Typhi strain. We generated an *ompR* deletion in *S.* Typhi Ty2, and this mutant also showed decreased infection of epithelial cells or macrophages (Figure 5).

**Figure 6:**
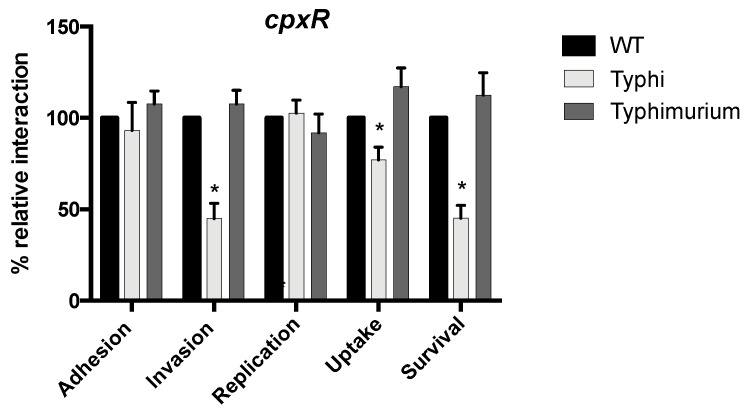
Comparison of the *cpxR* mutant of *S.* Typhi and *S.* Typhimurium. Epithelial INT-407 cells and THP-1 macrophages were infected with *S*. Typhi ISP1820 strain and *S.* Typhimurium SL1344 and their isogenic *cpxR* mutant. Both WT strains are settled at 100 percent. All assays were conducted in triplicate and repeated independently at least three times. The results are expressed as the mean ± SEM of the replicate experiments. Significant differences (* *p* < 0.0001) compared to the wild-type were determined by the Student’s unpaired *t*-test.

Then, as the *cpxR* mutant was found to be significantly less invasive than the wild-type strain in *S.* Typhi, but was able to invade and replicate in epithelial cells at levels comparable to the wild-type strain in *S.* Typhimurium [53,76], we constructed this mutant in *S.* Typhimurium SL1344 and investigated its interaction with cells (Figure 6). During interaction with epithelial cells, the *cpxR* mutant of *S.* Typhimurium was similar to the wild-type strain, suggesting that the effect is strain-specific to *S.* Typhi. There was also no difference between the wild-type and the SL1344 *cpxR* mutant when tested in the murine macrophages RAW264.7.

## 4. Discussion

TCS are usually the first to detect a perturbation in the intracellular or extracellular environment and will react quickly to modify bacterial gene expression. They are involved in sensing a variety of signals (pH, ions, nutrients, stress, etc.). Therefore, TCS are critical for bacterial adaptation and survival. Here, we have identified 30 TCS in the genome of *S.* Typhi and summarized their putative function and role in *Salmonella* (Table 1). We have deleted each of the TCS regulator encoding genes from *S.* Typhi and tested interactions with human epithelial cells (adhesion, invasion, and replication) and macrophages (uptake and survival), which constitute two important niches of *S*. Typhi infection. Moreover, these mutants represent important tools to advance our knowledge of *S.* Typhi pathogenesis by investigating their roles during interactions with cells or under different environmental conditions.

All the TCS mutants grew similarly to the wild-type strain in liquid culture. Most of the TCS mutants (24/30) showed a significant difference compared to the wild-type strain during at least one step of infection (adhesion, invasion, replication, uptake, or survival) (Table 2). There were 9 phenotypes previously associated with 8 TCS in *S.* Typhimurium that were confirmed in *S.* Typhi (*arcA*, *cheY*, *phoP*, *qseB*, *qseF*, *rcsB*, *sirA*, and *yehT*) (Table 2). Interestingly, several of the TCS previously associated with *S.* Typhimurium virulence in mice (*cheY*, *cpxR*, *narP*, *ompR*, *phoP*, *qseB*, *qseF*, *rcsB*, *sirA*, and *ssrB*) display a phenotype during host cell interaction with *S.* Typhi, except for *cpxR* and *ssrB*, see below (Table 2). An important aspect of this study was the identification of 32 new phenotypes associated with *S*. Typhi TCS mutants (Table 2). Interestingly, the *cpxR* mutant had phenotypes distinct from *S.* Typhimurium found in the literature (Table 2). The *S.* Typhimurium *cpxR* mutant was not affected for invasion or intracellular replication in epithelial cells (HEp2 and Caco-2) or survival in RAW264.7 macrophages [53], while the *S*. Typhi *cpxR* mutant was defective in invasion of INT407 cells and survival in THP-1 macrophages (Table 2). Thus, we have deleted *cpxR* in *S*. Typhimurium SL1344 and evaluated its level of adhesion, invasion, and replication in epithelial cells and in macrophages (Figure 6). No significant difference between the wild-type was observed, confirming a difference in the role of CpxR between *S.* Typhi and *S*. Typhimurium.

Six RR mutants, *creB*, *hydG*, *phoB*, *ssrB*, *ttrR* and *yehT* were similar to the wild-type strain in all conditions tested. The mutation of 4 TCS (*creB*, *hydG*, *phoB*, *ssrB*) in *S.* Dublin also resulted in a phenotype similar to the wild-type strain during infection of epithelial cells [110]. The deletion of the Ttr system of *S.* Dublin caused a higher level of invasion, but in *S*. Typhi, the *ttrS* sensor is a pseudogene (see below), which may explain why no phenotypes were observed. It may be surprising that the SsrAB system, which is the principal regulator of SPI-2, demonstrated no defect, but we have previously demonstrated that the entire SPI-2 deletion was not essential for *S*. Typhi survival in macrophages [23], and SPI-2 was not required for *S.* Typhi infection in a humanized mice model [111], highlighting one of the major differences with *S.* Typhimurium.

*S.* Typhi has evolved as a human-restricted pathogen without any known environmental niche. This specialization is associated with genome degradation, as up to 5% of its genome includes predicted open reading frames that have become pseudogenes. There are two TCS that are pseudogenes in *S.* Typhi: TorR and the sensor TtrS. The TtrSR system is involved in tetrathionate respiration in the inflamed gut, which provides a competitive advantage against the intestinal microbiota [112]. However, the production of the Vi capsule by *S.* Typhi prevents intestinal inflammation [9], suggesting that *S.* Typhi does not need the TtrRS system and the *ttrR* mutant did not show any phenotype in the tested conditions here. The TorSR system is not characterized in *Salmonella*. In *E. coli*, TorR activates the transcription of *torCAD* [113], which encodes proteins required for anaerobic respiration [114,115,116]. Here, even in the absence of a functional sensor, the *torR* mutant was defective in invasion and had a higher level of survival in macrophages.

Epithelial cell invasion was the infection step in epithelial cells where TCS mutants differed significantly when compared to the wild-type, as 16 mutants demonstrated changes in invasion (7 increased invasion and 9 decreased invasion). As expected, the *sirA* (*Salmonella* invasion regulator) deletion resulted in decreased invasion, consistent with the role of SirA in inducing SPI-1 [97,117]. The complementation of this mutant restored the wild-type level (Figure 3). By contrast, only 5 mutants were affected in their adhesion level and 8 in intracellular replication, compared to the wild-type. Interestingly, none of the TCS mutants had the same phenotypic pattern (Table 1), except for the 6 aforementioned mutants that did not differ from the wild-type. This emphasizes the diversity of TCS used to respond to environmental changes as well as the specificity of each system, as each TCS is unique.

The *rcsB* mutant showed increases in cell interactions for almost all tested conditions, except for intracellular replication in epithelial cells. RcsB belongs to the Rcs phosphorelay, a complex TCS with three members, RcsC, RcsD, and RcsB, and several accessory proteins involved in the stress envelope response. RcsB was shown to repress important virulence factors, including fimbriae, SPI-1, and also activation expression of the Vi capsule [16,90]. Thus, some virulence genes are expressed in the *rcsB* mutant, which lead to increased adhesion and invasion and the Vi capsule is repressed, which increased phagocytosis by these cells [118].

The *ompR* mutant showed the lowest level of adhesion and one of the lowest levels of invasion (Figure 1). These defects were restored by the addition of a wild-type copy of *ompR* (Figure 3). The motility of the *ompR* mutant was also reduced to 85% of the wild-type. An *ompR* mutant was attenuated in *S.* Typhimurium [71] and OmpR was associated with the activation of SPI-2 [73,74,75] and motility genes [119]. This regulation pattern is exactly the opposite of the Rcs system, which may explain why these mutants have strong and opposite phenotypes. These phenotypes are specific to each mutation and did not involve the Vi capsule.

## 5. Conclusions

Virulence genes expression needed to be tightly regulated in order for *S.* Typhi to adapt and survive within the host. TCS participate in the regulation of several virulence factors and we have shown that several TCS contribute to adhesion, invasion, replication, uptake, and survival of *S.* Typhi. Distinct phenotypes of the CpxR mutant of *S.* Typhi compared to *S.* Typhimurium may reveal fundamental regulatory differences associated with *S.* Typhi niche specialization. Further characterization of the regulons associated with TCS involved in virulence and identification of the signals required for their activation will be important to understand *S.* Typhi pathogenesis. This will help to identify and develop strategies to prevent and or to reduce typhoid infections.

## Figures and Tables

**Table 1:** Two-component systems of *Salmonella* Typhi and their putative function.

SK	RR	Function
ArcB (STY3507)	ArcA (STY4947)	Global aerobic respiration control; oxidative stress [38,39]; SPI-1 activation [40]; motility [41]; defective in invasion and survival [42]
BaeS (STY2343)	BaeR (STY2155)	Envelope stress: (antimicrobial resistance (AMR) and metal resistance) [43,44]
CitA (STY0062)	CitB (STY0061)	Anaerobic citrate fermentation^a^ [45]
CheA (STY2130)	CheY (STY2125)	Chemotaxis; required for virulence in mice [46], and in invasion [47]
CopS (STY1127)	CopR (STY1128)	Uncharacterized ^a^
CpxA (STY3813)	CpxR (STY3812)	Membrane stress (AMR and metal resistance) [48,49,50,51,52]; SPI-1 repression [53,54] and SPI-2 regulation [55]; virulence [53,55,56]
CreC (STY4936)	CreB (STY4935)	Carbon source metabolism ^a^ [57]
DcuS (STY4502)	DcuR (STY4501)	C4-dicarboxylates catabolism ^a^ [57,58,59]
DpiB (STY0674)	DpiA (STY0675)	SOS response ^a^ [60]
GlnL (STY3875)	GlnG (STY3876)	Nitrogen response [61,62]
HydH (STY3712)	HydG (STY3211)	Zinc transport ^a^ [63]
KdpD (STY0744)	KdpE (STY0743)	Potassium transport [64], *C. elegans* colonization [65]
NarX (STY1286)	NarL (STY1285)	Nitrate responsive [66,67]
NarQ (STY2718)	NarP (STY2472)	Nitrate respiration [66] ^a^; Involved in virulence [68]
EnvZ (STY4295)	OmpR (STY4294)	Envelope stress response. Osmolarity and acid resistance [69,70]; virulence [71], SPI-1 and SPI-2 control [72,73,74,75], Vi capsule activation [13,14]; adhesion and invasion [76]
PgtB (STY2634)	PgtA (STY2633)	Phosphoglycerate transport [77]
PhoR (STY0433)	PhoB (STY0432)	Inorganic phosphate assimilation [78]; SPI-1 regulation [79]
PhoQ (STY1270)	PhoP (STY1271)	Global virulence regulator [80]; Magnesium transport [81]; AMR [82,83,84]; invasion [85]; survival in macrophages [80]
PmrB (STY4490)	PmrA (STY4491)	LPS modification, AMR resistance, virulence [27,86]; SPI-2 repression [87]
QseC (STY3355)	QseB (STY3354)	Motility, invasion [27], macrophage survival [25], virulence [25,88,89]
QseE (STY2811)	QseF (STY2809)	Invasion and intramacrophage replication [25]; virulence [25]
RcsC (STY2496)	RcsB (STY2495)	Cell envelope stress response, Vi capsule activation [16]; virulence [90]; AMR [91]; LPS modifications; oxidative and acidic stress [92]; invasion [17]
RstB (STY1651)	RstA (STY1647)	Motility [93]; iron acquisition [94,95]
BarA (STY3096)	SirA (STY2155)	Virulence [96], SPI-1 [97], SPI-2 [97], motility [98], Vi capsule [99]; invasion [100]
SsrA (STY1728)	SsrB (STY1729)	SPI-2 regulator [11,101], oxidative stress [102]; SPI-1 repression [103]
TctE (STY2903)	TctD (STY2904)	Tricarboxylate transport [104]
TorS (STY3951)	*TorR* (STY3954)	Trimethylamine-N-oxide respiration (anaerobic) ^a^ [105]
*TtrS* (STY1735)	TtrR (STY1733)	Tetrathionate respiration [106]
UhpB (STY3993)	UhpA (STY3992)	Hexose phosphate transport [28], sulfur assimilation [29]
YehU (STY2389)	YehT (STY2388)	Poorly characterized, regulation of the carbon starvation protein (CstA) [107]

^a^ Role in Escherichia coli.

**Table 2:** Phenotype of regulator mutant during interaction with host.

	Epithelial Cells (INT407)			Macrophages (THP1)	
RR	Adhesion	Invasion	Rep	Uptake	Survial
ArcA		C		N	C
BaeR		N			N
CitB		N	N		N
CheY	N	C			N
CopR			N		N
CpxR		*		N	*
CreB					
DcuR					
DpiA					N
GlnG			N	N	
HydG					
KdpE				N	N
NarL				N	
NarP	N	N		N	N
OmpR	*	*		N	*
PgtA	N	N	N	N	
PhoB					
PhoP		C			C
PmrA		N	N		N
QseB		C	N		
QseF		C			
RcsB	N	C		N	N
RstA			N		
SirA		C			N
SsrB					
TctD		N			N
TorR		N			N
TtrR					
UhpA			N		
YehT		C			

Blue = significantly lower; Grey = no difference, Red = significantly higher than the wild-type. C = confirmed phenotype; N = new phenotype; * = divergent phenotype.

## Supplementary Materials

The following are available online at https://www.mdpi.com/2076-2607/8/5/722/s1: Table S1: Bacterial strains used in this study; Table S2: Plasmids used in this study; Table S3: Primers used in this study.
