# Supplementary material for: New Roles for Two-Component System Response Regulators of Salmonella enterica Serovar Typhi during Host Cell Interactions
Source: Microorganisms. 2020 May 13;8(5):722. doi: 10.3390/microorganisms8050722 (PMC7285189; doi:10.3390/microorganisms8050722)
Supplement: Supplementary file 1 [file microorganisms-08-00722-s001.pdf]

microorganisms

Table 1. Strains used in this study. Ajouter TviB, double mutant, complement, STM cpxR.

Strain	Description	Reference
S. Typhi ISP1820	wild type	(77)
DEF201	*ΔinvA ΔssrB*	This study
DEF433	χ8521, constitutive *phoP24*	R. Curtis III
DEF1239	*ΔarcA*	This study
DEF1248	*ΔbaeR*	This study
DEF1323	*ΔcitB*	This study
DEF1522	*ΔcheY*	This study
DEF1320	*ΔcopR*	This study
DEF1238	*ΔcpxR*	This study
DEF1254	*ΔcreB*	This study
DEF1306	*ΔdcuR*	This study
DEF1253	*ΔdpiA*	This study
DEF1307	*ΔglnG*	This study
DEF1318	*ΔhydG*	This study
DEF1240	*ΔkdpE*	This study
DEF1289	*ΔnarL*	This study
DEF1290	*ΔnarP*	This study
DEF1508	*ΔompR*	This study
DEF1523	*ΔpgtA*	This study
DEF570	*ΔphoB*	(82)
DEF1241	*ΔphoP*	This study
DEF1308	*ΔpmrA*	This study
DEF1312	*ΔqseB*	This study
DEF1310	*ΔqseF*	This study
DEF1319	*ΔrcsB*	This study
DEF1311	*ΔrstA*	This study
DEF1247	*ΔsirA*	This study
DEF149	*ΔssrB*	(30)
DEF1314	*ΔtctD*	This study
DEF1313	*ΔtorR*	This study
DEF1315	*ΔttrR*	This study
DEF1321	*ΔuhpA*	This study
DEF1322	*ΔyehT*	This study
DEF1511	*ΔompR* (pWSK:ompR)	This study
S. Typhi Ty2	wild-type	This study
DEF1516	Ty2Δ*ompR*	This study
S. Typhimurium SL1344	wild-type	(132)
DEF1479	SL1344 Δ*ompR-envZ*	This study
DEF1480	SL1344 Δ*cpxR*	This study
E. coli DH5αλpir	*supE*44 *hsdR*17 *recA*1 *endA*1 *gyrA*96 *thi*-1 *relA*1 λpir	Invitrogen
E. coli χ7213	Sm10 λpir *asd thi thr leu tonA lacY supE recA* RP4 2-Tc::Mu[λpir] Δ*asdA4*	(133)

Table 2. Plasmids used in this study (Ajouter complément et autres).

Plasmid	Description	Reference
pMEG375	*sac* RB, mobRP4, oriR6K, vecteur suicide	Megan Health
pSIF475	pMEG375 with flanking region of *arcA* gene used for *arcA* deletion	This study
pSIF480	pMEG375 with flanking region of *baeR* gene used for *baeR* deletion	This study
pSIF514	pMEG375 with flanking region of *citB* gene used for *citB* deletion	This study
pSIF553	pMEG375 with flanking region of *cheY* gene used for *cheY* deletion	This study
pSIF511	pMEG375 with flanking region of *copR* gene used for *copR* deletion	This study
pSIF476	pMEG375 with flanking region of *cpxR* gene used for *cpxR* deletion	This study
pSIF483	pMEG375 with flanking region of *creB* gene used for *creB* deletion	This study
pSIF484	pMEG375 with flanking region of *dcuR* gene used for *dcuR* deletion	This study
pSIF482	pMEG375 with flanking region of *dpiA* gene used for *dpiA* deletion	This study
pSIF485	pMEG375 with flanking region of *glnG* gene used for *glnG* deletion	This study
pSIF509	pMEG375 with flanking region of *hydG* gene used for *hydG* deletion	This study
pSIF477	pMEG375 with flanking region of *kdpE* gene used for *kdpE* deletion	This study
pSIF486	pMEG375 with flanking region of *narL* gene used for *narL* deletion	This study
pSIF487	pMEG375 with flanking region of *narP* gene used for *narP* deletion	This study
pSIF549	pMEG375 with flanking region of *ompR* gene used for *ompR* deletion	This study
pSIF554	pMEG375 with flanking region of *pgtA* gene used for *pgtA* deletion	This study
pSIF182	pMEG375 with flanking region of *phoB* gene used for *phoB* deletion	(82)
pSIF478	pMEG375 with flanking region of *phoP* gene used for *phoP* deletion	This study
pSIF489	pMEG375 with flanking region of *pmrA* gene used for *pmrA* deletion	This study
pSIF505	pMEG375 with flanking region of *qseB* gene used for *qseB* deletion	This study
pSIF497	pMEG375 with flanking region of *qseF* gene used for *qseF* deletion	This study
pSIF510	pMEG375 with flanking region of *rcsB* gene used for *rcsB* deletion	This study
pSIF498	pMEG375 with flanking region of *rstA* gene used for *rstA* deletion	This study

pSIF479	pMEG375 with flanking region of *sirA* gene used for *sirA* deletion	This study
pSIF074	pMEG375 with flanking region of *ssrB* gene used for *ssrB* deletion	(30)
pSIF506	pMEG375 with flanking region of *tctD* gene used for *tctD* deletion	This study
pSIF507	pMEG375 with flanking region of *torR* gene used for *torR* deletion	This study
pSIF508	pMEG375 with flanking region of *ttrR* gene used for *ttrR* deletion	This study
pSIF512	pMEG375 with flanking region of *uhpA* gene used for *uhpA* deletion	This study
pSIF513	pMEG375 with flanking region of *yehT* gene used for *yehT* deletion	This study
pSIF548	pWSK29 with *ompR*	This study

Table 3. Primers used in this study.

Gene	Primers	Sequence
arcA	ArcA F1	cgggatccCCCACGACCAAGCTAATGAT
	ArcA R2	CCGTGAATGATAAGAATGTGCGGGGTCTG
	ArcA F3	CATTCTTATCATTCACGGCGAAGGTTATC
	ArcA R4	gctctagaGTCCTGTGAGCATCCCCTTA
baeR	BaeR F1	cgggatccACGGAAGTGTCCCGTAACC
	BaeR R2	CGTATAAATCTGTCCCAGCTTGGGTTCAT
	BaeR F3	CTGGGACAGATTTATACGCGCGGTCTACG
	BaeR R4	gctctagaTAGCGGTGAGATGACGTTTC
citB	CitB F1	cgggatccATTTTCGAGCGTGGAGTGAC
	CitB R2	TAGCTGATCGCTAGCATCGGTTCGTCTTC
	CitB F3	GATGCTAGCGATCAGCTACGGGAAAGTGG
	CitB R4	gctctagaACCCTGCAATCCTGTTTTGT
cheY	cheY F1	cgggatccCGAAGCAAGTTGTGTGGTGT
	cheY R2	GTTTCTCAAGCATGGTCGAAAAGTCATCC
	cheY F3	CGACCATGCTTGAGAAACTGGGCATGTGA
	cheY R4	gctctagaCCGTCATCTGGACGACATAA
copR	CopR F1	cgggatccTCGTTTCATGCTTCACTCCTT
	CopR R2	ACTTTGGCGCCCTGACGTACCCACTCAAT
	CopR F3	ACGTCAGGGCGCCAAAGTAGACGATCCAT
	CopR R4	gctctagaGCCCGATTTATTAGCGTCAT
cpxR	CpxR F1	cgggatccGCCATAACAGCAGCGGTAAC
	CpxR R2	AAACCACGGCAGCTCTCGGTCATCATCAA
	CpxR F3	CGAGAGCTGCCGTGGTTTAAAACATTGCGT
	CpxR R4	gctctagaTCTCTACGCGGCCATATTTT
creB	CreB F1	cgggatccCAGGGAGAGGTGGTTTTCAA
	CreB R2	TAGTTTAGCTCCCTGTTCATCCTCGACTA
	CreB F3	GAACAGGGAGCTAAACTACGCGCGATCA
	CreB R4	gctctagaGGTTGCCGGAAAGGAGAT
dcuR	DcuR F1	cgggatccGGTTACGGTGCTGATTACGG
	DcuR R2	GCTGAACGCCGACATAGCGGCGATTTAAC
	DcuR F3	GCTATGTCGGCGTTCAGCCGGAACATTAC
	DucR R4	gctctagaCACTCTCGGCATGGGATAAT
dpiA	DpiA F1	cgggatccAAGTACCCGAGCTGACGAAC
	DpiA R2	AATCAGGTGGCGGATATATTCTGCGTGCAT
	DpiA F3	TATATCCGCCACCTGATTGTCGCAGAGAT
	DpiA R4	gctctagaCCGGGATGGCTAAAATATCA
glnG	GlnG F1	cgggatccGCTGTTCTACCCGATGGTCA
	GlnG R2	ACCCTGCGTCACCCAACGGATGGAACTAT
	GlnG F3	CGTTGGGTGACGCAGGGTCATAAACAGGA
	GlnG R4	gctctagaCGCTCCACTCGATACCAGAT
hydG	2-HydG R4	gctctagaTGGCTGACGGTAAGGTTTTC
	2-HydG F3	CGCTGCTTCTGGAGAAAGAGGTGATTCTGG
	2-HydG R2	CTTTCTCCAGAAGCAGCGCCTGTAAAATC
	2-HydG F1	cgggatccGAACGCTATTCATGCGATTG
kdpE	KdpE F1	cgggatccATAATGGGCCGGGTATTCCT
	KdpE R2	GGCGTGGGCGGATGGCCTGTTCATCTTCA
	KdpE F3	AGGCCATCCGCCCACGCCATTTTATTATC
	KdpE R4	gctctagaGCAATCTGTGAACCAGATCAAC
narL	NarL F1	cgggatccAAACGCCGAACGCAGTAAT
	NarL R2	CCAGACGCCTTACACCCGTGCGTAGCAT
	NarL F3	CGGGTGTAAGGCGTCTGGATATCACCGAAA
	NarL R4	gctctagaCAGCGTGTCTCTTCCAATGA
narP	NarP F1	cgggatccGCAGCTACATATGCCACACT
	NarP R2	AGCCCCTGCATACCTCGCCGCATAAGTGG
	NarP F3	GCGAGGTATGCAGGGGCTTTCTAACAAAC

	NarP R4	gctctagaTGGCGCAGGAGAAATAAGAC
ompR	OmpR F1	cgggatccggggttgccgattaattgta
	OmpR R2	ACCGTCCGGACGCATATCGTCATCAACCA
	OmpR F3	GATATGCGTCCGGACGGTTCTAAAGCA
	OmpR R4	gctctagaCAGTTGCAGCTTATCGGT
pgtA	PgtA F1	cgggatccGCGCTGGAGGTTTATATTGC
	PgtA R2	TTTGCAAGTCCGCTTTTACCCACTCTTTG
	PgtA F3	TAAAAGCGGACTTGCAAATTCCCCGTAA
	PgtA R4	gctctagaTGTCAAATCGTCGGACAAGA
phoP	PhoP F1	cgggatccTGACGCCGGCAAATTATATC
	PhoP R2	CGTGCGGATTCAGGTGGTGGCGTAATAA
	PhoP F3	ACCACCTGAATCCGCACGATGTCATTACC
	PhoP R4	gctctagaGCAGACGAAACGTGGTTTTA
pmrA	PmrA F1	cgggatccACATGTCCCGATGCTCATTT
	PmrA R2	GTTCGTTATGCGTGTCGTCTTCAACAATC
	PmrA F3	ACGACACGCATAACGAACCCTCGACCAA
	PmrA R4	gctctagaTTTGCTCAGTGCTTTCATGC
qseB	QseB F1	aaggaaaaaagcggccgcTCCCTTTCATGTCGTTTCAG
	QseB R2	TTCCACTGCACGTCCCTCGGTAAACCAGT
	QseB F3	GAGGGACGTGCAGTGGAAGTCCATGTTCA
	QseB R4	gctctagaGGCGAACAACATCAACTGTG
qseF (yfhA)	YfhA F1	cgggatccTGGGGAGTTTAACCGACATT
	YfhA R2	GAGAGTAGCTTCAGTAATCCGGGATCGTC
	YfhA F3	ATTACTGAAGCTACTCTCCCGTCACGAA
	YfhA R4	gctctagaCTTCAGTGACCGTCATACCG
rcsB	RcsB F1	cgggatccGGGTGTTTGCCATGCTTAAT
	RcsB R2	ATTTCTTCTCAAGTGATTTGCGAATACCG
	RcsB F3	AATCACTTGAGAAGAAATCGGCGATGATG
	RcsB R4	gctctagaCTGTTTGTCGAAGCCTGTGA
rstA	RstA F1	cgggatccGGGCAGCTTTAATCACGAGT
	RstA R2	TCAGCAGCGTAAGCGGCAATGAGAGAACC
	RstA F3	TGCCGCTTACGCTGCTGAGCCCTATCGTAT
	RstA R4	gctctagaAAGGGGCTGTTCCGACTAAT
sirA	SirA F1	cgggatccAAATAGCAGCCCGGAACAG
	SirA R2	CTCCGCATTCACCAGTTCGTGGTCATCAA
	SirA F3	GAACTGGTGAATGCGGAGACGTTAACAAGC
	SirA R4	gctctagaATGCGTTACCGTGACATCAA
tctD	TctD F1	aaggaaaaaagcggccgcTGCTTGCAGTAAGGGTACGA
	TctD R2	CTTCCCTGAGCCAGCTCACGGTTATCTTC
	TctD F3	TGAGCTGGCTCAGGGAAGCGATGTACGAAT
	TctD R4	gctctagaAGGGCACGTTAACTTCCAGA
torR	TorR F1	cgggatccAACGCTCTCCAGAGTTTTCG
	TorR R2	AAGCGGCGAGATACCCCTCCTGCTCAAAA
	TorR F3	AGGGGTATCTCGCCGCTTACGTCATAAAAT
	TorR R4	gctctagaCAACCGGTGCCTGAAAATA
ttrR	TtrR F1	cgggatccATTACGTCAGGCGTTTCAGC
	TtrR R2	AATCAGTTCCGCCGTATCATCATCCAGTA
	TtrR F3	GATACGGCGGAACTGATTAGGCGTTTCG
	TtrR R4	gctctagaATCTCGCGGTACAGTTCGTC
uhpA	UhpA F1	cgggatccGGAACCTTGCTTCAACTGGA
	UhpA R2	ACCGCCATATCGCAGATACACACCTGTACG
	UhpA F3	TATCTGCGATATGGCGGTAAAAGAGATTGC
	UhpA R4	gctctagaAGGCCGAAGGGAAAGAGTAG
yehT	YehT F1	cgggatccTAATGCGGGACTGTATCAGC
	YehT R2	GCGACGGCTTAACGGCTCATCATCCACAA
	YehT F3	GAGCCGTTAAGCCGTCGCTATCTGAAAA
	YehT R4	gctctagaGCTGAAACCCCTCTTCCTCT

ompR-EnvZ	ompR-EnvZ F1	cgggatccGGGGTTGCCGATTAATTGTA
	ompR-EnvZ R2	tgtcgtcccACGCATATCGTCATCAACCA
	ompR-EnvZ F3	gatatgcgtGGGACGACAAAAGAGGCATA
	ompR-EnvZ R4	gctctagaTGGCGAAACTGTTCATTGAG
